# Malaria's Missing Number: Calculating the Human Component of *R_0_* by a Within-Host Mechanistic Model of *Plasmodium falciparum* Infection and Transmission

**DOI:** 10.1371/journal.pcbi.1003025

**Published:** 2013-04-18

**Authors:** Geoffrey L. Johnston, David L. Smith, David A. Fidock

**Affiliations:** 1 Department of Microbiology and Immunology, Columbia University College of Physicians and Surgeons, New York, New York, United States of America; 2 School of International and Public Affairs, Columbia University, New York, New York, United States of America; 3 Bloomberg School of Public Health, John Hopkins University, Baltimore, Maryland, United States of America; 4 Division of Infectious Diseases, Department of Medicine, Columbia University College of Physicians and Surgeons, New York, New York, United States of America; Emory University, United States of America

## Abstract

Human infection by malarial parasites of the genus *Plasmodium* begins with the bite of an infected *Anopheles* mosquito. Current estimates place malaria mortality at over 650,000 individuals each year, mostly in African children. Efforts to reduce disease burden can benefit from the development of mathematical models of disease transmission. To date, however, comprehensive modeling of the parameters defining human infectivity to mosquitoes has remained elusive. Here, we describe a mechanistic within-host model of *Plasmodium falciparum* infection in humans and pathogen transmission to the mosquito vector. Our model incorporates the entire parasite lifecycle, including the intra-erythrocytic asexual forms responsible for disease, the onset of symptoms, the development and maturation of intra-erythrocytic gametocytes that are transmissible to *Anopheles* mosquitoes, and human-to-mosquito infectivity. These model components were parameterized from malaria therapy data and other studies to simulate individual infections, and the ensemble of outputs was found to reproduce the full range of patient responses to infection. Using this model, we assessed human infectivity over the course of untreated infections and examined the effects in relation to transmission intensity, expressed by the basic reproduction number *R_0_* (defined as the number of secondary cases produced by a single typical infection in a completely susceptible population). Our studies predict that net human-to-mosquito infectivity from a single non-immune individual is on average equal to 32 fully infectious days. This estimate of mean infectivity is equivalent to calculating the human component of malarial *R_0_*. We also predict that mean daily infectivity exceeds five percent for approximately 138 days. The mechanistic framework described herein, made available as stand-alone software, will enable investigators to conduct detailed studies into theories of malaria control, including the effects of drug treatment and drug resistance on transmission.

## Author Summary

We report a new mathematical model of the progression, within a human host, of a malaria infection caused by the parasite *Plasmodium falciparum*. This model incorporates probability distributions for the key parameters of infection and transmission so that model outputs match the entire range of observed responses in patients, without the requirement for fitting individual data. Further, we simulate the daily densities of both the disease-causing and transmissible forms of the parasite within an individual, as well as the onset of fever and the probability of parasite transmission to mosquitoes. This model allows us to reproduce aspects of infection that are critical for malaria control modeling. As a first application, we calculate the net infectiousness of humans to mosquitoes and predict that net human infectivity from a single infection is on average equal to approximately 32 fully infectious days. This value has been used to help map the worldwide intensity of malaria transmission. We also predict that mean daily infectivity is greater than five percent for approximately 138 days. Our modeling framework, available as downloadable software, will allow researchers to probe the effects of treatment and drug resistance on malaria transmission in unprecedented detail, helping to improve malaria control efforts.

## Introduction

Approximately 2.5 billion people live in areas whose local epidemiology permits transmission of *Plasmodium falciparum*, the parasite that causes the most life-threatening form of malaria [1]. Malaria has inflicted a severe toll in morbidity and mortality over the course of human history. Nonetheless, recent studies, however, document significant reductions in malaria mortality over the past decade [2], [3]. Given these encouraging results, public health experts are planning campaigns to reduce or eliminate transmission from many areas of the world [4], [5]. To help assess the feasibility of eliminating malaria from an area, efforts are ongoing to model and map the historical and current limits of this transmission. These models and maps also help establish a baseline to judge the success of these efforts [1], [6], [7]. The development of these mathematical frameworks, however, is complicated by the diversity of mosquito vectors, varying levels of human immunity, and the extent to which control efforts are applied.

The development of mathematical models of malaria is contingent on a detailed understanding of the parasite lifecycle. This begins in humans when motile parasite forms, termed sporozoites, enter the body through the bite of an *Anopheles* mosquito and travel to the liver where they rapidly proliferate. Upon emerging from the liver, parasites then enter the blood stream as merozoites. These merozoites infect red blood cells (RBCs), develop, replicate, burst from the infected cells, and repeat the cycle of asexual blood stage infection that causes disease. These asexual blood stages are able to avoid clearance in the spleen by expressing surface ligands that enable parasitized red blood cells (PRBCs) to adhere to endothelial cells in the microvasculature [8], [9]. This property of cytoadherence and sequestration results from surface expression of *P. falciparum* erythrocyte membrane protein (PfEMP1). Because PfEMP1 presents a prominent antigenic target for the immune system, *P. falciparum* has evolved a sophisticated system of epigenetically-regulated antigenic variation, whereby individual parasites typically express only a single, antigenically-distinct member of the multigene family *var* that encodes PfEMP1 [8], [9]. Expression continuously switches between *var* genes as a mechanism to continually present new epitopes that escape an already existing antibody response. Separate from the pathogenic asexual blood stages, intra-erythrocytic parasites can also differentiate into sexual stages known as gametocytes [10]. Once parasites have committed to becoming gametocytes, they sequester in the bone marrow or microvasculature and develop through four stages for 7–12 days [11]. They then reenter the circulation to complete their maturation as Stage V gametocytes. Mature Stage V male and female gametocytes are then primed to form gametes and mate in the midgut of the definitive host, the *Anopheles* mosquito, following blood meal ingestion.

Many models of malaria have been developed to describe this cycle of transmission from the mosquito to the human host and back. These models can be broadly classified into two categories: compartmental and mechanistic. A compartmental model is any type of transmission model that simulates populations of individuals transitioning into different compartments at constant rates, with each compartment representing a different state of disease/non-disease. For example, an “SIR” model is a compartmental model in which individuals are grouped into three populations, namely susceptible (S), infectious (I), and recovered (R). Individuals transition between compartments at a constant rate depending on several factors that include the virulence of the disease and the immune responses of hosts. More sophisticated models include additional compartments that each represent a different disease state. For example, the infective compartment may be separated into multiple compartments (I_1_, I_2_, I_3_, etc.) each with different levels of infectiousness, or other compartments may be added, for example infected but not infectious hosts (known as the E compartment) [12]. The basic units of analysis in compartmental models are populations; the number of individuals within each disease state is tracked over time, but individuals are categorized only to the extent that they occupy one of the various compartments.

In contrast, mechanistic malaria models incorporate the within-host mechanisms that determine human infectiousness over time. In such models, individual hosts are the primary units of analysis [13], [14]. Transitioning among different levels of infectivity occurs because of individual clearance of infections, and parasite densities are modeled at the individual level. Individuals differ in multiple parameters including the intensity and duration of infection and the timing of fever.

Each of these two frameworks has a useful role to play. Compartmental models benefit from simplicity, identifiably, and clarity, while mechanistic ones allow for simulations of control measures that are highly non-linear. Regardless of the model type, one of the most important mathematical quantities for theories of disease control aimed at elimination is *R_0_*, the basic reproduction number [15]. *R_0_* is defined as the number of secondary cases that an index case would generate in a population without previous exposure to the disease. *R_0_* serves as a threshold criterion for transmission: if the *R_0_* of an area is below 1, the disease will eventually become extinct; if above 1, the disease will spread. For malaria, *R_0_* can be expressed as the product of the vectorial capacity (the number of infectious mosquito bites that result from mosquitoes taking blood meals on a fully infectious human in a single day), the duration of the human infectious period, and the efficiency of transmission from humans to mosquitoes.

Vectorial capacity can be estimated using a variety of techniques [7], [16]–[22]. However, the human component of malaria transmission is difficult to quantify, in part, because the transmissibility of an infection is affected by many competing factors. Although a variety of mathematical models have been built to simulate the progression of malaria infections, [13], [23]–[27], no model has yet produced an estimate of net human infectivity over time.

Here, we report a stochastic, mechanistic, within-host model that simulates the progression of *Plasmodium falciparum* infections and human-to-mosquito infectivity. We built upon previously published work by Molineaux and Dietz, who first developed the asexual and gametocyte components of our model from malaria therapy data, in which individuals with tertiary syphilis were infected with *P. falciparum* to induce a fever and clear the syphilitic bacteria [23]–[25]. This framework has been used to simulate the effects of vaccines on transmission [13], [23]–[26]. However, this earlier work required that parameters be fitted to an individual patient's case history before simulation. We have extended these earlier studies by choosing stochastic distributions for model parameters, thereby allowing for within-host simulations that generate an ensemble of infection dynamics that are consistent with observed malaria therapy infections. We also included additional components that enable simulations of human-to-mosquito infectivity and onset of symptoms.

Using this model, we have examined the levels of human-to-mosquito infectivity over time and isolated the host-related determinants of the basic reproduction number, *R_0_*. This novel analysis of *R_0_* made it possible to analyze overlooked aspects of transmission relevant for elimination campaigns. We calculated that net human infectivity is equivalent to 32 fully infectious days, on average. Further, we calculated the distribution of infectiousness within human populations given the natural variability of individuals' immune responses to infection, as well as the mean infectivity of a population over time. We found that infectiousness from malaria persists for a long duration of time: mean infectiousness is predicted to exceed five percent for 138 days after infection. These results were then compared to outputs from compartmental models [12], [28], [29]. We propose that the modeling work described herein provides the most careful estimate yet of the distribution of human responses to malaria infection and the mean human contribution to *R_0_*. Our model also provides a framework to examine how antimalarials may affect malaria transmission, given the complexities of host-parasite dynamics.

## Methods

### Defining a model of asexual parasitemia

The model used to calculate asexual parasitemia is a within-host model that simulates the course of an infection one replication cycle after merozoites have emerged from the liver. Asexual parasitemias are modeled as a system of discrete (two-day time interval) difference equations previously elaborated by Molineaux et al. [13], [23]. The model simulates parasite densities in 50 different subpopulations differentiated by *var* gene expression type. In each replication cycle, a fixed percentage of parasites in each subpopulation switch into a different population. The switching probabilities are structured such that certain *var* genes are expressed with higher probability than others; immune pressure against variants also plays a role (the switching phenomenon is described below). Asexual parasitemia densities are regulated by three host immune responses: an innate response that establishes an upper limit for parasite density; a PfEMP1 variant-specific response that regulates short-term periodic oscillations in density; and a variant-transcending response that causes a steady log-linear decrease in density over time, clearing the infection. We do not simulate deaths from malaria, as these are so few in proportion to the very large number of total infections as to not significantly impact overall transmission.

Our model was fitted to data from malaria therapy patients, in which individuals with tertiary syphilis and with no acquired immunity to malaria were inoculated with single strains of *P. falciparum* in order to induce a fever and clear the infection [30], [31] (see details below). Thus, our asexuals model best reproduces the time course of asexual parasitemias in malaria-naïve adult male patients who exhibited effective immune responses.

#### 1. Quantities modeled


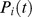
: The number of red blood cells infected by *Plasmodium falciparum* parasites displaying PfEMP1 type *i* at time *t*


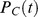
: The cumulative number of number of red blood cells infected at time *t*

#### 2. Constants and parameters

*s*: Proportion of isotype population that switches *var* expression in each period; constant

*v*: Number of PfEMP1 variants (set to 50); constant

*M*: Minimum parasitemia simulated by model; constant


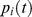
: Probability that isotype population *P_j_* will switch into population *P_i_*; variable, see description below in section on antigenic variation

#### 3. Equations determining asexual parasitemia.



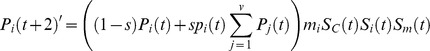


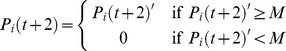


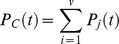



### Host immune response parameters

Our within-host model incorporates three types of immune responses. An innate response 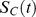 represents inflammation, fever, and cytokine responses to parasite replication and is a function of total parasite load, irrespective of PfEMP1 type. The two other immune responses are acquired and are dependent on antibody production. 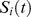 represents the PfEMP1 variant-specific immune response, with the response to each isotype denoted by the subscript *i*. 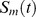 represents the acquired PfEMP1 variant-transcending immune response. This immune response is provoked by the conserved regions of PfEMP1 (since some PfEMP1 variants have been shown to induce cross-reactivity) as well as conserved surface proteins (such as MSP-1) and other parasite antigens. Both of the antibody responses are assumed to decay exponentially over time in the absence of new antigen production in our model.

Each of these three responses has a characteristic effect on the progression of parasitemia. The innate response controls the initial densities of asexual parasitemia and is dominant during the initial period of infection. The second response, the variant-specific acquired response, controls the characteristic peaks and dips in parasitemia and interacts with the *var* switching structure to determine the densities of PfEMP1 variants over time. The third response is the variant-transcending acquired response, which produces the roughly log-linear decline in parasitemias over time and helps to clear the infection. We assumed that the strengths of the innate and variant-transcending immune responses vary among individuals.

#### 1. Quantities modeled


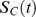
: The innate immune response


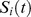
: The PfEMP1 variant-specific acquired response


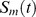
: The PfEMP1 variant-transcending acquired immune response

#### 2. Constants and parameters


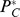
: Determines the parasite density at which the innate immune response reaches 50% of maximum; stochastic (see below)


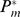
: Determines the parasite density at which the PfEMP1 variant-transcending immune response reaches 50% of maximum; stochastic (see below)


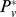
: Determines the parasite density at which the PfEMP1 variant-specific immune response reaches 50% of maximum; constant

*k_C_*: Determines functional form (Hill slope) of innate immune response to total parasitemia; constant

*k_m_*: Determines functional form (Hill slope) of variant-transcending immune response to cross-reactive epitopes; constant

*k_v_*: Determines functional form (Hill slope) of variant-specific immune response to individual PfEMP1 variants; constant

*σ*: Decay rate of acquired immune response to PfEMP1 variant; constant

*δ_v_*: Delay in onset of acquired immune response to PfEMP1 variant; constant

*τ*: Index variable used to sum over previous asexual parasitemia levels

*β*: Affects levels of acquired variant-transcending immune response; constant

*δ_m_*: Delay in onset of variant-transcending acquired immune response; constant

*ρ*: Decay rate of variant-transcending acquired immune response; constant

*C*: Level of parasitemia above which variant-transcending immunity does not increase; constant


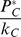
: Asexual parasite density at the first peak of parasitemia (the maximum asexual parasitemia); stochastic following a truncated log-normal distribution with a mean of 10^4.79^ parasites per µL (this was the median among malaria therapy patients from [23]) and a scale parameter of 1.148 (from [32])


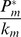
: First asexual parasitemia observation day minus the last asexual parasitemia observation day; stochastic following a Gompertz distribution (as reported in [33]) with shape parameters (0.0311, 0.0004) chosen to fit the malaria therapy data from Sama et al. [33]

#### 3. Equations determining host immune functions.



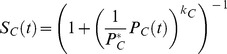


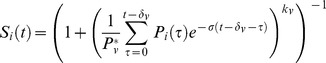


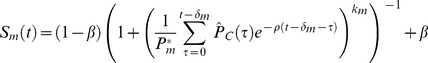


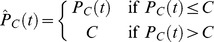



### Antigenic variation

In our model, PfEMP1 variant densities are explicitly modeled. The parasite population is partitioned into 50 different subpopulations, each representing one antigenic isotype (*i*). The probability that a given isotype population *P_j_* will switch into the population *P_i_* is given by the probability *p*_i_(*t*), which changes over time (and is independent of *j*). The probability *p*_i_(*t*) is designed to incorporate three aspects of *var* switching leading to expression of the antigenically distinct PfEMP1 proteins.

First, we assume that the PfEMP1 status of parasites is reset during the mosquito stage such that infections start with a single PfEMP1 variant [34]. Second, we assume that the probability of switching into variants is not constant among the variants, but is structured such the likelihood of switching into some variants is greater than the likelihood for others [34]–[36]. This pattern may reflect the distance of the *var* genes from the telomeric regions or other types of inherent *var* structure and gene regulation [34]–[36]. Third, we assume that a PfEMP1 variant has a decreased probability of appearing if the immune system has previously mounted a response to that variant. The biological rationale for this assumption is that a prior immune response will decrease the probability of a variant appearing because parasites expressing this variant are more likely to be cleared before reaching densities detectable by smear.

It is also possible that more than one variant, even most or all variants, are expressed at the onset of infection [37], [38]. However, the innate response controls the initial phase of infection (before antibodies are developed), and this response is independent of the PfEMP1 types present. During this early period of infection, there is likely to be selective pressure from the host against some isotypes, such that some isotypes are eliminated [38]. Thus, even if all variants are expressed initially, only some will survive to the period when antibodies are formed. The difference between a model in which all variants are expressed initially and our model is that the former relies entirely on variant cross-reactivity and/or immunodominance to maintain infections [39], [40], whereas ours relies on both cross-reactivity and the appearance of less likely variants to maintain infections.

In our model, we also assume that parasites expressing different PfEMP1 variants proliferate at different rates. We assign each isotype a growth rate *m_i_* chosen from a truncated normal distribution. This assumption is based on experimental evidence that some PfEMP1 variants proliferate faster than others *in vivo* (specifically, some variants that are associated with severe disease have been shown to propagate faster than those that are not) [41]. Further, certain variants may be better adapted to a host's particular biology than others, resulting in differences in net growth rates *in vivo* [38].

#### 1. Quantities modeled


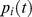
: Probability that a PRBC will switch to the *i*th PfEMP1 isotype at time *t*+2; units are in probability of switching per two days

#### 2. Constants and parameters

*q*: Parameter for geometric distribution affecting isotype-dependent switching probability; constant (set to 0.3)

*I*: Minimum immune response provoking *var* switching; constant (set to 0.1)


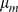
, 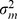: Parameters for normal distribution describing isotype-specific growth rates; constant

#### 3. Equations describing PfEMP1 dynamics.



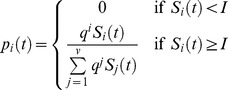

*m_i_*: Growth rates of different PfEMP1 variants; stochastic with distribution 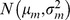 truncated so that 1≤*m_i_*≤35

### Modeling the onset of first fever

Because we are interested in utilizing this model to simulate drug treatment in low-transmission areas, treatment-seeking behavior is an important consideration. In the absence of diagnostic testing, fever may serve as an indicator of infection for both patient and clinician [42], [43]. To predict when fever first occurs, we utilize modeling by Dietz et al. [44] who used malaria therapy data to fit probability distributions to the onset of fever. In our model, all patients are assumed to be symptomatic and to experience a fever that begins a variable number of days before reaching maximum parasitemia. To determine the day of first fever following one cycle of replication after emergence of parasites from the liver into the blood stream (taken as time zero), we use a uniform distribution based on an individual's maximum asexual parasitemia [13], [44].

Specifically, we simulate the time course of an individual infection from inoculation to clearance and record the maximum parasitemia achieved (denoted 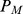). We then take a random draw (denoted *d*) from the distribution *U*(log_10_(0.0002), 0) = *U*(−3.699, 0) and calculate 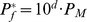 [13], [44]. The first day that an individual's parasitemia is greater than or equal to 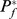 is assumed to be the first day of fever for that individual.

#### 1. Quantities modeled

*feverday*: The predicted first fever day of an individual, set to the first day that an individual's asexual parasitemia is greater than or equal to 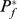; variable, depends on individual simulation


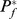
: Fever threshold; variable, depends on individual simulation

#### 2. Constants and parameters

*F*: Lower limit of uniform distribution determining fever threshold; constant (set to 0.0002)


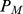
: Maximum level of asexual parasitemia; variable, depends on individual simulation

#### 3. Equations determining first fever


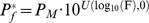
, where *U*(*a*, *b*) is a draw from the uniform distribution with lower bound *a* and upper bound *b*

### Modeling gametocyte densities over time

Our gametocytemia model equations were first articulated by Diebner et al. and Eichner et al. (the two models differ slightly; we adhere to the formulation by Eichner et al.) [24], [25]. In our model gametocytes are produced by each wave of asexual parasitemia at a stochastic frequency determined by the function γ(). Gametocytes are assumed to sequester for a variable number of days as they develop. Once the mature gametocytes emerge into the blood stream, they are cleared by the immune system, die naturally, or are transmitted to a mosquito. Our gametocyte model simulates levels of circulating (Stage V) gametocytes as well as numbers of gametocytes in the earlier stages (Stages I–IV) on a daily timescale. Gametocyte lifetimes, in the absence of immune response related to asexual parasitemia, are assumed to follow a Gompertz distribution [24]. We assume that the degree of anti-gametocyte immunity is related to the cumulative levels of previous asexual parasitemia. We do not include any suppressive effect of fever on gametocyte densities, as reported in [45].

As in the asexual model described above, the original gametocyte modeling work [24], [25] fitted model parameters to each individual patient's malaria therapy data. We modified their model by choosing model parameters from probability distributions such that the resulting outputs matched the observed variability in the malaria therapy data.

#### 1. Quantities modeled

*G*(*t*): The number of mature gametocytes circulating in the bloodstream. Gametocyte gender is not modeled (see section on infectivity below).

#### 2. Constants and parameters

*D_S_*: Sequestration time for gametocyte maturation in days; stochastic with truncated normal distribution (*μ* = 7; *σ* = 1.5); the truncation interval is set so that 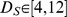


γ: Asexual to sexual conversion probability, peak specific; stochastic following log-normal distribution with location parameter of −6 and a scale parameter of 4 in natural log space

*α_G_*: Rate at which age affects gametocyte mortality; stochastic with uniform distribution between .06 and 1

*β*: Effects of previous levels of asexual parasitemias on gametocyte death rates; constant

*μ_0_*: Initial age-related component of total gametocyte mortality rate; constant

*τ*: Index variable used to sum over gametocyte ages (in number of days old)

#### 3. Equations determining gametocyte density.



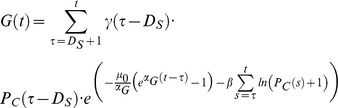



### Human-to-mosquito infectivity parameters

In the original Ross-Macdonald model, the infectivity of humans to mosquitoes was parameterized by a constant, *c* [46]. In our model we estimate the probability of human-to-mosquito transmission (defined as production of an oocyst [47]) as a function of gametocyte levels. For our baseline simulations, we utilize the nonlinear relationship between gametocytemia and infectivity described by Stepniewska et al. based on mosquito feeding studies on malaria therapy patients [47]–[49]. Net infectivity for an individual is quantified by taking the area under a curve generated from predicted infectivity over time; this quantity is equivalent to the number of fully infectious days.

Our model of infectivity is not mechanistic in the same way that our asexual and gametocyte models are. We use the sigmoidal curve reported in [48] to force high gametocyte densities to be substantially less infectious than would be predicted by a proportional model of infectivity. We do not model why this nonlinearity occurs. Two main hypotheses could explain the reduced infectivity of gametocytes at high densities. The first is that gametocytes themselves regulate their infectiousness in a density-dependent manner [50]–[53] such that high densities are proportionally much less infectious than low densities. The second hypothesis is that host factors (antibodies, cytokines, fever) affect the infectivity of gametocytes [27], [54]–[57], though fever was not found to influence the infectivity of gametocytes in malaria therapy [58]. In our model, we do not include these possible additional factors in the calculation of human-to-mosquito infectivity; however, we did conduct a sensitivity analysis to examine the effects of different density-to-infectivity relationships on our model outputs.

A final note regarding infectivity is that of Jeffery and Eyles in their original 1955 study of mosquito feedings on malaria therapy patients [47]. These authors observed that, in the first two to four days after gametocytes were observable in the bloodstream of infected patients, individuals were not infectious to mosquitoes. They attributed this phenomenon to the fact that, when gametocytes are first becoming microscopically detectable, they are immature and are thus unable to infect mosquitoes. We account for the observed non-infectivity of gametocytes appearing very early in the course of infection by adjusting modeled infectivity profiles slightly. Specifically, if the difference between the first observable asexual and sexual parasitemias was 15 days or less for a simulated individual, then this individual becomes infectious two days after gametocytes were first observed. For individuals with larger differences between asexual and gametocyte patency, or that never have an observable gametocytemia, we assume that individuals are not infectious until more than 17 days after asexual blood stage parasites are first detectable. This adjustment roughly corresponds to the feeding study data reported by Jeffery and Eyles [47].

#### 1. Quantities modeled

*c*(*x*): The infectivity of humans to mosquitoes (i.e. the percent chance that a mosquito blood meal will result in oocysts in the mosquito midgut), where *x* is the gametocyte density per µL.

#### 2. Constants and parameters

*mintrans*: Minimum gametocyte density that allows for transmission

#### 3. Equations determining gametocyte infectivity.
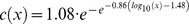


The functional relationship between gametocyte density and infectivity used here is taken from [48]. Note that *c*(*x*) is set to 0 for a variable period during the onset of gametocyte appearance (as reported in [47]). Our default model also assumes that infectivity is 0 if gametocyte density is below 2/3 gametocytes per µL, due to the need for 2 gametocytes to be present per blood feed (assuming 3 µL of blood per feed); we call this threshold *mintrans*. We vary these assumptions in the sensitivity analysis.

### Fitting our model to malaria therapy data

#### Prior information on parameter distributions

In the initial formulation upon which we built our asexuals model [23], two parameters (infection duration and maximum density) were fitted to individual patient case histories. To develop our mechanistic model, we found distributions from the published literature to inform our choice of both parameters. For the duration of infection model parameter, a study found that the durations of infection in malaria therapy were Gompertz distributed with a mean duration of patent parasitemia of 210.7 days [33]. For the distribution of maximum parasitemias, a previous within-host model parameterized to malaria therapy data used a log-normal distribution [32]. For the mean of this distribution, we used the median (10^4.79^ asexual parasites per µL) observed among 35 malaria therapy patients [23]; to set an upper bound, we used the maximum observed parasitemia (10^5.66^ asexual parasites per µL) from [23].

In the original formulation of our gametocyte model, five parameters were fitted to individual patient case histories [24], [25]. After a literature review we found data that determined two of the five distributions. Eichner et al. reported that the sequestration delay *D_S_* roughly followed a normal distribution [25] with a mean of 7.4 days. Eichner et al. also reported that 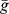, the average asexual-sexual conversion probability, roughly followed a lognormal distribution with a mean of 0.0064; this information informed our choice of γ, the asexual-sexual model conversion distribution. The other three gametocyte model parameters (*α_G_*, *β*, and *μ_0_*) had insufficient information to determine their distributions.

#### Fitting asexual parasitemias and gametocyte densities

The first component that we fitted to data was the asexuals model. For our simulation target data, we used the distribution of durations of infection from malaria therapy [59] as well as the minimum, median, and maximum of 9 clinical indicators from malaria therapy data. These malariometric indices were derived by Molineaux et al. from 35 malaria therapy patient charts [23]. We note that these 35 charts were selected from a total of 334 patients because this subset was classified as ‘spontaneous cures’ given their treatment history (although some of the 35 did receive low-dose suppressive treatments). Note also that we defined a ‘local maxima’ in asexual parasitemia as a parasitemia a) greater than the 6 values preceding it and b) greater or equal to the 6 values following [23].

With the target data defined, we then fitted the model to these data. For a measure of the goodness-of-fit, we used the relative errors between model outputs and the min, median, and max of the 9 indices, as well as the distances between the modeled and observed durations of infection (as measured from the cumulative distribution functions). We used the log-normal distribution for maximum parasitemias with a mean of 10^4.79^ asexual parasites per µL to set 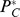, which determines the parasite density at which the innate response reaches 50% of maximum [23], [32]. To fit the duration of infectivity and the min, med, and max of the nine indices from malaria therapy, we varied *k_C_*, which determines the relationship between asexual density and level of innate immune response, *σ*, the decay rate of the variant-specific response, and 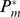, which determines the parasite density at which the variant-transcending response reaches 50% of maximum. We also truncated the isotype-specific growth rates *m_i_* to have a maximum value of 35 [23]. Choosing these four parameters to fit to data allowed us to decrease the overall model degrees of freedom but still have control over all three types of immune response.

To fit the gametocyte model parameters we first needed to define our target data. We used *D_S_*, the average duration of gametocyte sequestration, 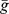, the average asexual to sexual parasite conversion probability, and *L*, the average length of time that gametocytes are observed in the circulation, as our target indices. These indices were derived for the malaria therapy data by Eichner et al. [25]. For a measure of the goodness-of-fit, we used the differences between the geometric mean, minimum, and maximum values from malaria therapy and model outputs. When calculating gametocyte densities from asexual parasitemias we assumed that asexual parasitemias were local maxima only if they satisfied the two criteria above as well as were c) greater than or equal to 100 PRBC/µL (as in [25]).

We had three gametocyte model parameters to fit for which we had insufficient prior information: *α_G_*, *β*, and *μ_0_*. As for the asexual model, there were too many degrees of freedom to test model outputs against malaria therapy data using all possible combinations of model parameters. We used the reported quantiles for these three parameters from Diebner et al. [24] to help inform our choice of initial values. After experimenting with model outputs, assuming a variety of different distributions for the parameters, we found that we could reproduce the range of observed variation with *β* and *μ*_0_ being fixed at their population means (as reported in [24]) and *α_G_* varying according to a uniform distribution. **Table 1** provides the best-fit values and distributions for these asexual and gametocyte model parameters; parameters that are not listed in **Table 1** remain unchanged from their previously published values [23]–[25].

**Table 1:** Best-fit model parameter constants and distributions.

Model	Parameter	Reference value	Revised value/distribution^3^	Description
Asexual	*k_c_*	0.2^1^	0.164	Affects levels of innate immune response to total parasitemia
Asexual	σ	0.02^1^	0.15	Decay rate of acquired immune response to PfEMP1 variant
Asexual	P_c_*/*k_c_*	Fitted to case history 1	truncated ln*N*(μ,σ^2^), *μ* = ln(10^4.79^), *σ* = 1.148, truncation point = 5.5	Asexual parasite density at the first peak of parasitemia
Asexual	P_m_*/*k_m_*	Fitted to case history 1	Gompertz(*α*,*θ*), *α* = 0.0311, *θ* = 0.0004	First day with observed asexual parasitemia minus last observed day
Asexual	*m_i_*	truncated *N*(*μ*,*σ* 2), μ = 16, σ = 10.4, truncation point = 1^1^	truncated *N*(*μ*,*σ* 2), *μ* = 16, *σ* = 10.4, truncation points = 1, 35	Growth rates of different PfEMP1 variants
Gametocyte	*D_s_*	Fitted to case history^2^	round(truncated *N*(*μ*,*σ* 2)), *μ* = 7, *σ* = 1.5, truncation points = 4, 12	Sequestration time for gametocyte maturation
Gametocyte	*γ*	Fitted to case history 2	truncated ln*N*(*μ*,*σ* 2), *μ* = −6, *σ* = 4, truncation point = 0.189	Asexual to sexual conversion probability, peak specific
Gametocyte	*α_G_*	Fitted to case history 2	*U*(0.06,1)	Rate at which age affects gametocyte mortality
Gametocyte	*β*	Fitted to case history 2	0.0013	Effects of previous asexual parasitemias on gametocyte death rates
Gametocyte	*μ_0_*	Fitted to case history 2	0.03	Initial age-related component of total gametocyte mortality rate

1 See ref [23].

2 See ref [24].

3 The best-fit parameters for the asexual and gametocyte components of our mechanistic model are shown for those parameters that have been modified from their original values [23], [24]. All other model parameters not provided above remain equal to their values in [23], [24]. ‘Fitted to case history’ indicates that the model was run with this parameter as a free parameter and the best-fit value was chosen after fitting outputs to the case history of an individual treated using malaria therapy.

#### Assessing the goodness-of-fit

We first consider the goodness-of-fit of the asexual component of the model. **Table 2** compares the minima, medians, and maxima of the malaria therapy data to bootstrapped values from the model outputs using the best-fit parameters. From this table, we see that the geometric means of the temporal intervals between local maxima are shorter in the model than observed in the data (**Table 2**, index 2–5). In other words, the ‘peaks’ of asexual parasitemia occur closer together in the model than in the data (though the total number of peaks in the model and the data are approximately the same). This difference may indicate that some *var* switching rates need to be reduced in our model, or that shared epitopes among variants repress densities for longer *in vivo* than in the current model. Further, the model slightly overestimates the mean proportion of positive observations in both halves of patency, indicating that the model predicts that infections are more often observable during their duration of patency than are observed clinically (**Table 2**, indices 2-7, 2-8). The model also overestimates the variability of the height of the peaks associated with PfEMP1 variants (**Table 2**, index 2-6). The model does fit data quite well for density at first maximum, as well as last positive day, with very low relative errors for those indices (**Table 2**, indices 2-2, 2-9).

**Table 2:** Comparison of asexual model outputs to malaria therapy data.

Index	Index description	Minimum observed from malaria therapy patients 1	Median observed from malaria therapy patients	Maximum observed from malaria therapy patients	Minimum model values 2	Median model values	Maximum model values	Relative errors, difference of minima	Relative errors, difference of medians	Relative errors, difference of maxima
2-1	Initial slope	0.19	0.49	0.87	0.24	0.52	0.75	−0.26	−0.07	0.14
2-2	Log density at first local maximum	3.37	4.79	5.66	3.69	4.78	5.67	−0.09	0.00	0.00
2-3	Number of local maxima	2	10	17	2	9	17	−0.21	0.06	−0.01
2-4	Slope of local maxima	−0.074	−0.013	−0.001	−0.091	−0.015	−0.007	−0.22	−0.12	−9.51
2-5	Geometric mean of the intervals between consecutive local maxima	14.4	20.0	77.8	1.5	14.6	28.4	0.90	0.27	0.64
2-6	Standard deviation of the logs of the consecutive local maxima	0.03	0.20	0.47	0.04	0.31	0.56	−0.31	−0.56	−0.20
2-7	Proportion of positive observations in the first half of the interval between first and last positive day	0.4	0.88	1.0	0.57	0.97	1.0	−0.43	−0.10	0.00
2-8	Proportion of positive observations in the second half of the interval between first and last positive day	0.08	0.46	0.94	0.12	0.58	1.00	−0.48	−0.27	−0.06
2-9	Last positive day	37	215	405	38	193	404	−0.02	0.10	0.00

1 See ref [23]. Nine malariometric indices were calculated from malaria therapy data [23]. The minimum, median, and maximum values for each of these indices are from 35 malaria therapy patients.

2 Indices from best-fit model outputs. To calculate these indices from model outputs, we used a bootstrapping procedure. The mechanistic malaria model was run 1,000 times and 50 samples of 50 runs each were selected. The number of runs (50) per sample was chosen to match the sampling procedure in [23]. For each sample, the minimum, median, and maximum value for each index was chosen; these indices were then averaged over all samples. The end time for all runs was 800 days.

For the gametocyte model, we were able to set the delay of appearance parameter *D_S_* directly given prior information [25], and so there is little error between modeled and observed mean, minimum, and maximum values. For the observed average asexual-to-sexual conversion probability 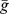, this is driven mostly by the parameter γ, and so we were also able to match the observed variation with little absolute error (**Table 3**). The average length of time that gametocytes are circulating in the bloodstream (index *L*) is controlled in the model by the immune parameters *α_G_*, *β*, and *μ_0_*. By setting *β* and *μ_0_* to their population means and allowing *α_G_* to vary uniformly between 0 and 1, we could generate the entire range of malaria therapy variation. However, having individuals with average gametocyte circulation times of 22 days yielded a model that was difficult to reconcile with other data sets [60]. We thus set *α_G_*∼*U*(0.06, 1) such that the maximum average gametocyte circulation time was set to be approximately 14 days (**Table 3**).

**Table 3:** Comparison of gametocyte model outputs to malaria therapy data.

	*D_S_*		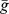		*L*	
	Malaria therapy^1^	Simulated^2^	Malaria therapy	Simulated	Malaria therapy	Simulated
Minimum	4.0	4.0	2.7 E-04	6.5 E-05	1.3	3.2
Geometric Mean	7.4	6.9	0.0064	0.0066	6.4	5.6
Maximum	12.0	10.8	0.135	0.111	22.2	13.9

1 See ref [25]. Three properties of within-host gametocyte dynamics were imputed from malaria therapy data. The properties are *D_S_*, the gametocyte sequestration time in days; 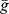, the average gametocyte conversion probability; and *L*, the length of time gametocytes persist in circulation. The first column for each parameter lists the value from 113 malaria therapy patients [25].

2 Gametocyte properties were calculated from the mechanistic malaria model outputs using best-fit gametocytemic parameters. Model values are from 50 samples of 113 runs each, from a total of 1,000 runs. The end time for all runs was 800 days.

Of note, we did not explore the entire parameter space for the asexual and gametocyte models, given computational limitations. Our final parameters were chosen as best-fits when the model outputs were qualitatively judged to be acceptably close according to the goodness-of-fit described above. We thus cannot provide precise point estimates with confidence intervals for our parameters. Nevertheless, sensitivity analyses were performed for certain parameters, as described below.

As a further check of our model outputs, we also compared our model outputs to other data not explicitly used in the model fitting. The modeled arithmetic mean duration of time between first fever and first gametocytemia detectable by smear among gametocyte-positive individuals was ∼12.9 days. This compares closely to the measured value from malaria therapy patients (10–11) [45]. Also, Jeffery and Eyles in their original 1955 study of mosquito feedings on malaria therapy patients reported that gametocytes generally become observable 10–15 days after parasite patency [47]. We found similar values (mode ∼11 days; median ∼11 days), although the model also generated larger values (∼20% of gametocyte positive individuals had differences between the first day of asexual patency and the first day of gametocyte patency ≥20 days).

## Results

### Development of a mechanistic model of within-host malarial infection

We have developed a mechanistic model of the progression of malaria within a human host, parameterized such that the model reproduces the median and extremes of the dynamics of infection observed in malaria therapy. For the asexuals model, we fitted five model parameters to the minimum, medians, and maxima of nine different malariometric indices derived from malaria therapy data. For the gametocyte model, we fitted five model parameters to the minima, geometric means, and maxima of three different indices derived from the gametocytemias of malaria therapy patients. **Table 1** illustrates those model parameters that were changed from published reports. A mathematical formulation of the model, as well as a description of how it was fitted to data, is described in the Methods. Standalone versions of the model for Macintosh or PC platforms are provided in the Supporting Information (see *MACmodel.zip* and *PCmodel.zip*), along with user manuals (**Text S1**) and an illustration of the graphical user interface (**Figure S1**).

**Figure 1:**
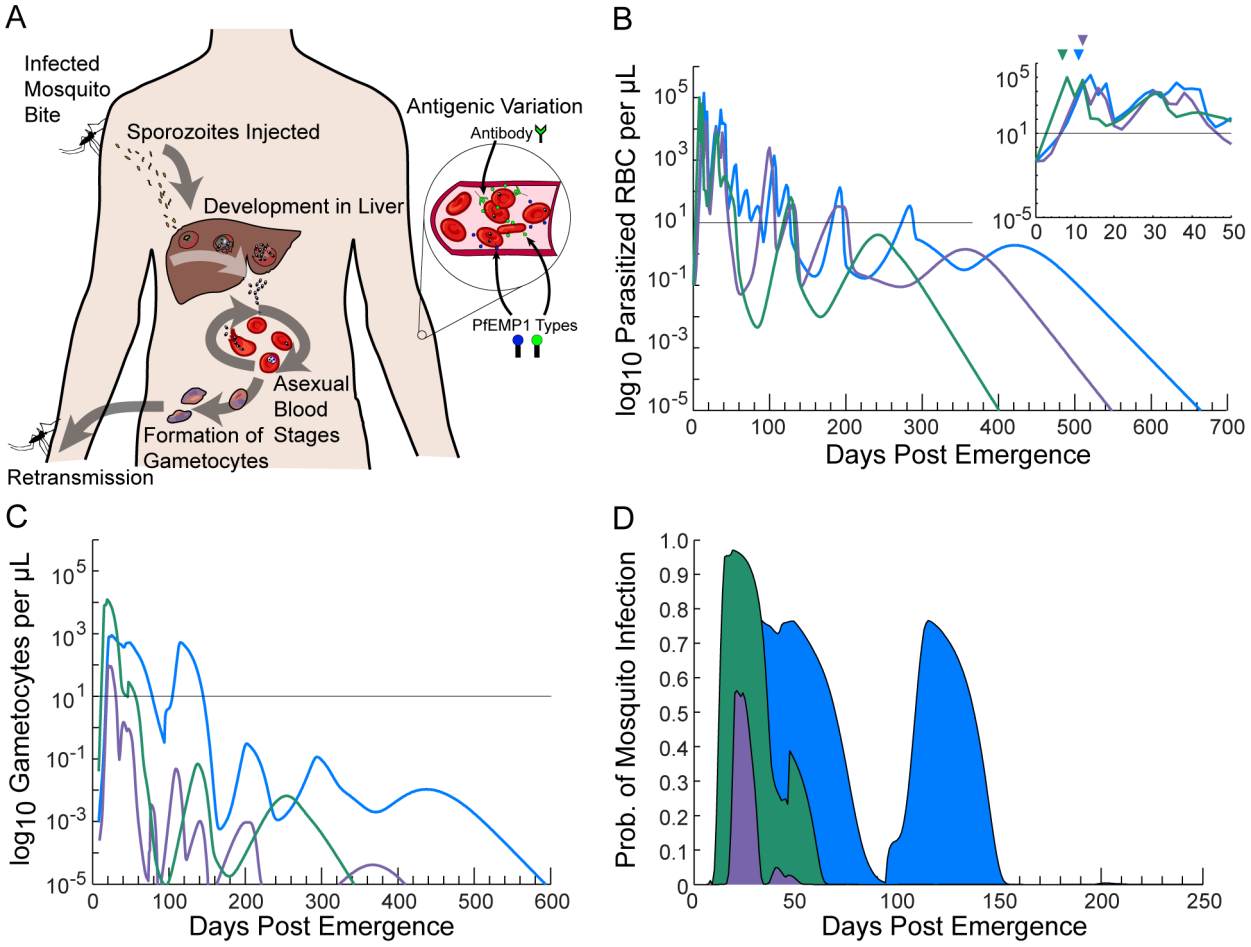
Illustration of asexual, sexual, and infectivity outputs. Our mechanistic *P. falciparum* infection model was used to simulate three individuals' host-parasite dynamics. (A) Schematic representation of the *P. falciparum* life cycle. The parasite is transmitted to humans though the bite of an infected mosquito. Motile forms (sporozoites) travel to the liver where they proliferate as liver stage parasites that and are then released into the blood. Parasites then adopt ∼48 hr cycles of red blood cell (RBC) invasion, asexual blood stage replication, and egress. Some intra-erythrocytic parasites differentiate into sexual forms (gametocytes) for uptake by further mosquito bites. Asexual parasites avoid immune capture by antigenic variation, primarily PfEMP1 cycling. (B) Individual log_10_ asexual parasitemias presented as a function of the number of days post emergence of parasites from the liver into the bloodstream. The inset depicts the first 50 days of infection; triangles above indicate the first day of fever. The black line is the level of detectability by microscopy (10 parasitized red blood cells (PRBC)/µL). (C) Daily gametocytemias of the same three individuals. (D) Estimated probability of human-to-mosquito transmission. Areas under the infectivity curves are equivalent to the number of fully infectious days. Although the model predicts the persistence of long-lived low-level and sub-detectable infections (as observed in malaria therapy), this panel illustrates how the bulk of infectivity usually occurs early in the course of infection.

**Figure 1** graphically illustrates the important features of our model by presenting three individual simulations. **Figure 1A** illustrates the *P. falciparum* lifecycle for reference. **Figure 1B** shows simulated asexual parasite densities over time, expressed as log_10_ PRBC per µL of blood. The black line illustrates the lower limit of detectability by microscopy (10 PRBC/µL) [45], [61]. The individual depicted in green has patent parasitemias for a period of ∼50 days, lapses into sub-patent parasitemia for ∼60 days, then has a short period of patency before relapsing permanently into sub-patent parasitemias. The infection is completely cleared by ∼day 400, post emergence of parasites from the liver. The characteristic peaks and dips apparent in the densities are associated with PfEMP1-based antigenic variation. The individual in purple displays three separate periods of patent parasitemia, whereas the individual in blue also has four periods, with the first lasting nearly 100 days. The inset in **Figure 1B** shows the first 50 days of infection along with the first fever day for each individual (onsets of fever are indicated by triangles). Fever is simulated to occur on day 7 post emergence for the individual depicted in green, day 11 for the blue individual, and day 12 for the purple.

**Figure 1C** shows the daily gametocytemias of the simulated individuals from **Figure 1B**. Note that the x-axis scale has been reduced from 700 to 600 for clarity. For the green individual, ∼10% of the first wave of asexual parasites converts to gametocytes. However, the later waves of asexual parasitemia have much lower asexual-to-sexual conversion probabilities, resulting in sub-patent gametocytemias after ∼day 60 and essentially no gametocytes after day 340. The asexual-to-sexual conversion probability is chosen stochastically for each wave of asexual parasitemia for each individual according to the distribution observed from malaria therapy (the geometric mean probability of conversion is approximately 0.7%). For the blue individual, the first asexual wave has a lower conversion probability than the second, resulting two gametocyte peaks of roughly equal height; gametocytes disappear from microscopic detection near day 140 and are completely cleared by day 600. For the purple individual, conversion probabilities are so low that gametocytes are patent only for a very short period between days 20 and 40 post emergence and are cleared completely near day 400.

**Figure 1D** illustrates the daily probabilities of human-to-mosquito transmission (i.e. the probabilities that a mosquito bite on these individuals would produce oocysts). The x-axis scale is now reduced from 600 to 250 days. To calculate the infectivity curves in **Figure 1D**, the gametocyte densities in **Figure 1C** were transformed using a sigmoidal relationship derived from feeding studies on malaria therapy volunteers [48] (see section below on gametocyte densities and their relationship to human-to-mosquito infectivity). Net infectivity is calculated by integrating the daily human-to-mosquito infectivity curves over time (shaded areas). The peaks of patent gametocytemia for the green, blue, and purple individuals in **Figure 1C** are clearly mirrored in **Figure 1D**, though the peaks of infectivity are exaggerated due to the transformation from density to infectivity.

### Assumptions concerning antigenic variation

As illustrated in **Figure 1**, an important feature of within-host malaria dynamics is antigenic variation. This variation is governed to a considerable extent by the nature of *var* gene switching leading to the expression of antigenically distinct PfEMP1 variants. In our model, we assumed that *var* is reset during infection so that only one variant is expressed after emergence from the liver. We then assumed that a fixed percentage of parasites switch into a new *var* type per replication cycle, with certain *var* variants more likely to appear than others. Further, we assumed that immune pressure against a given variant would reduce its likelihood of appearing.

**Figure 2:**
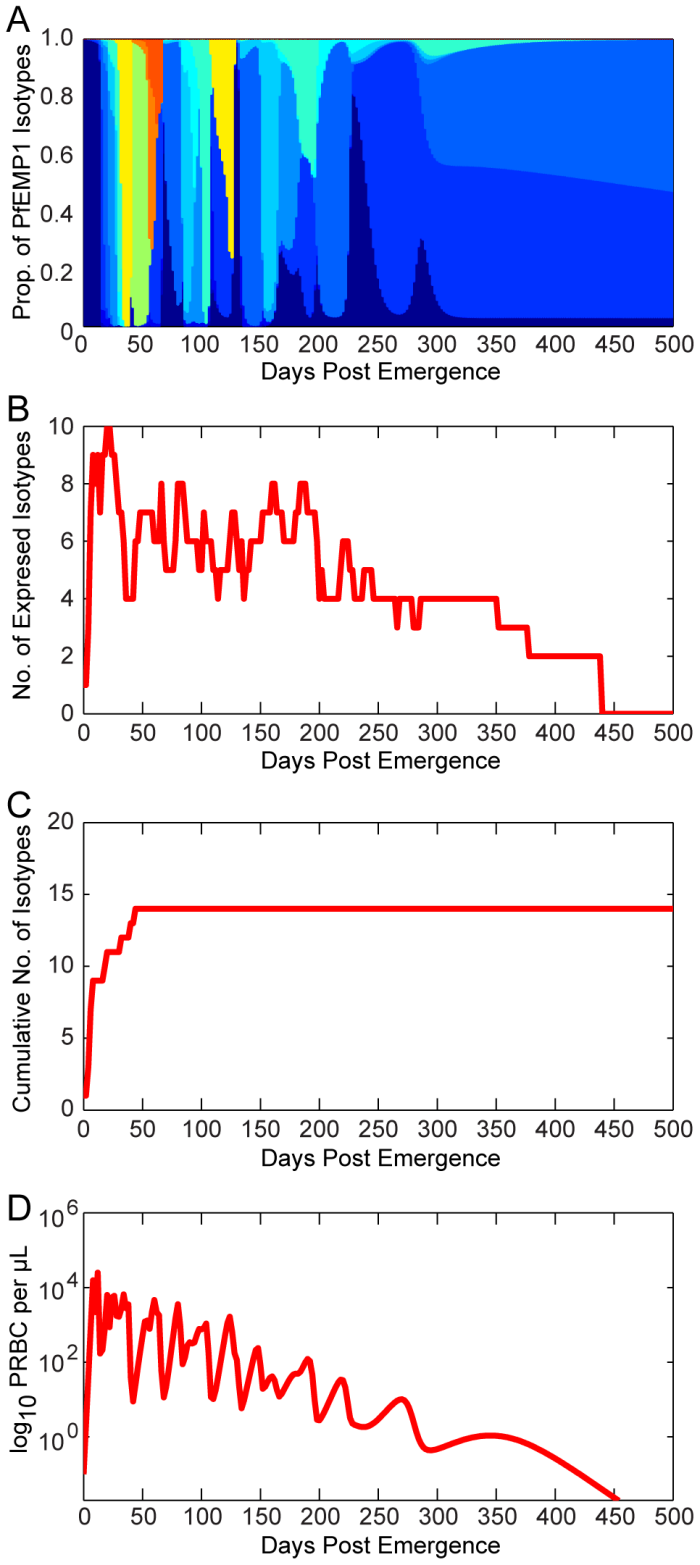
Illustration of model ***var*** **dynamics.** The members of the PfEMP1 family of *P. falciparum* erythrocyte membrane proteins are encoded by *var* genes, present at ∼60 copies per genome and each expressing a different PfEMP1 type. (A) Total asexual parasitemia as a function of time post emergence from the liver was modeled and the proportion filled by each PfEMP1 variant is shown in a different color. The number of colors and their respective levels at a given time indicates the diversity of isotypes present. Results are shown from a single model output. (B) The number of isotypes circulating in the blood over time. Isotypes are ‘expressed’ only if the density of that isotype is greater than or equal to 0.02 parasitized red blood cells (PRBC) per µL (the assumed threshold for PCR detection). (C) The cumulative number of isotypes that have been expressed over time (modeled from a single infection). (D) Levels of total asexual parasitemia over time for the illustrated run, in log_10_ PRBC per µL.

**Figure 2** illustrates the *var* (PfEMP1) expression patterns for a representative simulated individual. **Figure 2A** decomposes the total parasitemia over time into the various *var* subpopulations, such that each color corresponds to the proportion of parasitemia for each given type. Individual *var* types are counted as expressed only if their corresponding parasite populations reach 0.02 parasites per microliter, the assumed threshold for detection by polymerase chain reaction [62]. **Figure 2B** shows the total number of *var* variants expressed at any given time post emergence, and **Figure 2C** shows the cumulative number of *var* variants that have been expressed during the course of the infection (some variants are removed by the immune response). This particular simulation has a maximum of 10 variants simultaneously expressed within the first few days of infection, and this level decreases over time because of immune clearance. Because the switch rates for some variants are assumed to be faster than others (following a geometric series with a common ratio of 1/3), simulations exhibit a substantial *var* variation early in the infection, with only a few less-favored variants appearing later. **Figure 2D** illustrates the total parasitemia over time, which is affected not only by the *var* switch rate but also by the three types of host immune response (innate, variant-specific, and variant-transcending).

### Relationships between gametocyte density and parasite infectivity to mosquitoes

**Figure 3:**
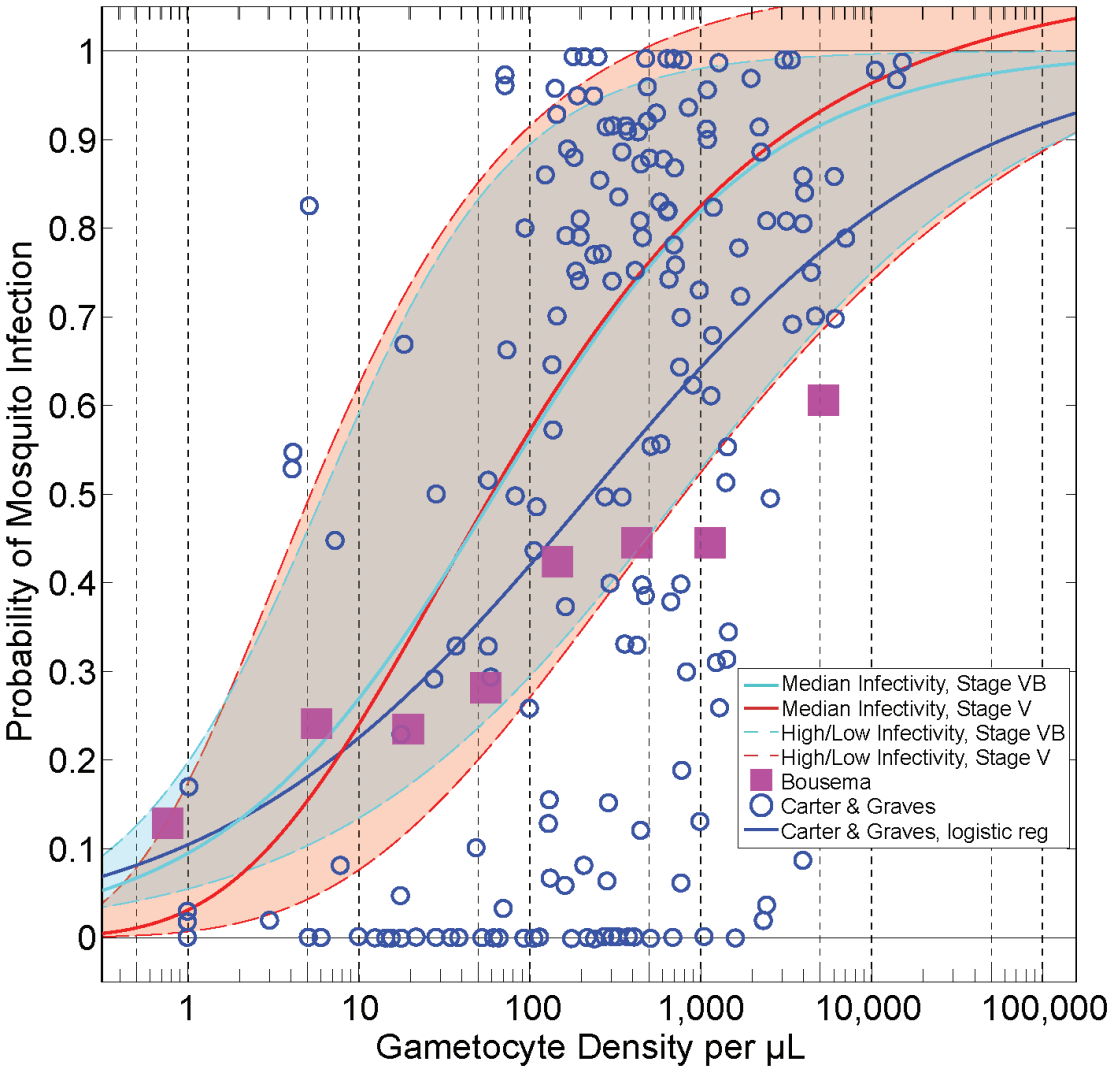
Relationships between gametocyte density and probability of human-to-mosquito infectivity. The scatterplot data (blue circles) were collated by Carter and Graves from multiple studies [63], [64]. The blue line is a logistic regression through the Carter and Graves data. The Bousema data indicate the relationship between infectivity and density from skin feeding studies with predominantly African volunteers in endemic settings [56]. The red line indicates the infectivity of malaria therapy volunteers (‘Median Infectivity, Stage V’) [48]; this parameterization is assumed to be the default. The red dotted lines illustrate the ‘High’ (maximum) and ‘Low’ (minimum) infectivity curves used in the model. The light blue solid and dotted lines indicate the relationships between gametocyte density and infectivity, assuming only Stage VB gametocytes are infectious (see Methods). All infectivity relationships included in the model are truncated at 1 (i.e. 100% probability of infection).

Another important determinant of human infectivity is the assumed relationship between gametocyte density and parasite infectivity to mosquitoes (also referred to as human-to-mosquito infectivity). A variety of functions relating gametocytes to infectivity have been described and proposed in the literature [48], [56], [63], [64]. All of these relationships share two features: a) infectivity increases monotonically with density, and b) high gametocyte densities are proportionally less infectious than low densities. However, the exact shapes of the curves differ. For our best-fit parameterization, we relied upon the functional form fitted by Stepniewska et al. [48] from human feeding studies conducted from malaria therapy patient volunteers. **Figure 3** illustrates this sigmoidal relationship (in red, denoted ‘Median Infectivity, Stage V’), as well as a scatterplot of density versus infectivity data from Carter and Graves [63], [64] (blue circles) and from a meta-analysis by Bousema et al. [56] (purple squares).

### Goodness-of-fit of modeled durations of asexual infection and gametocyte densities

**Figure 4:**
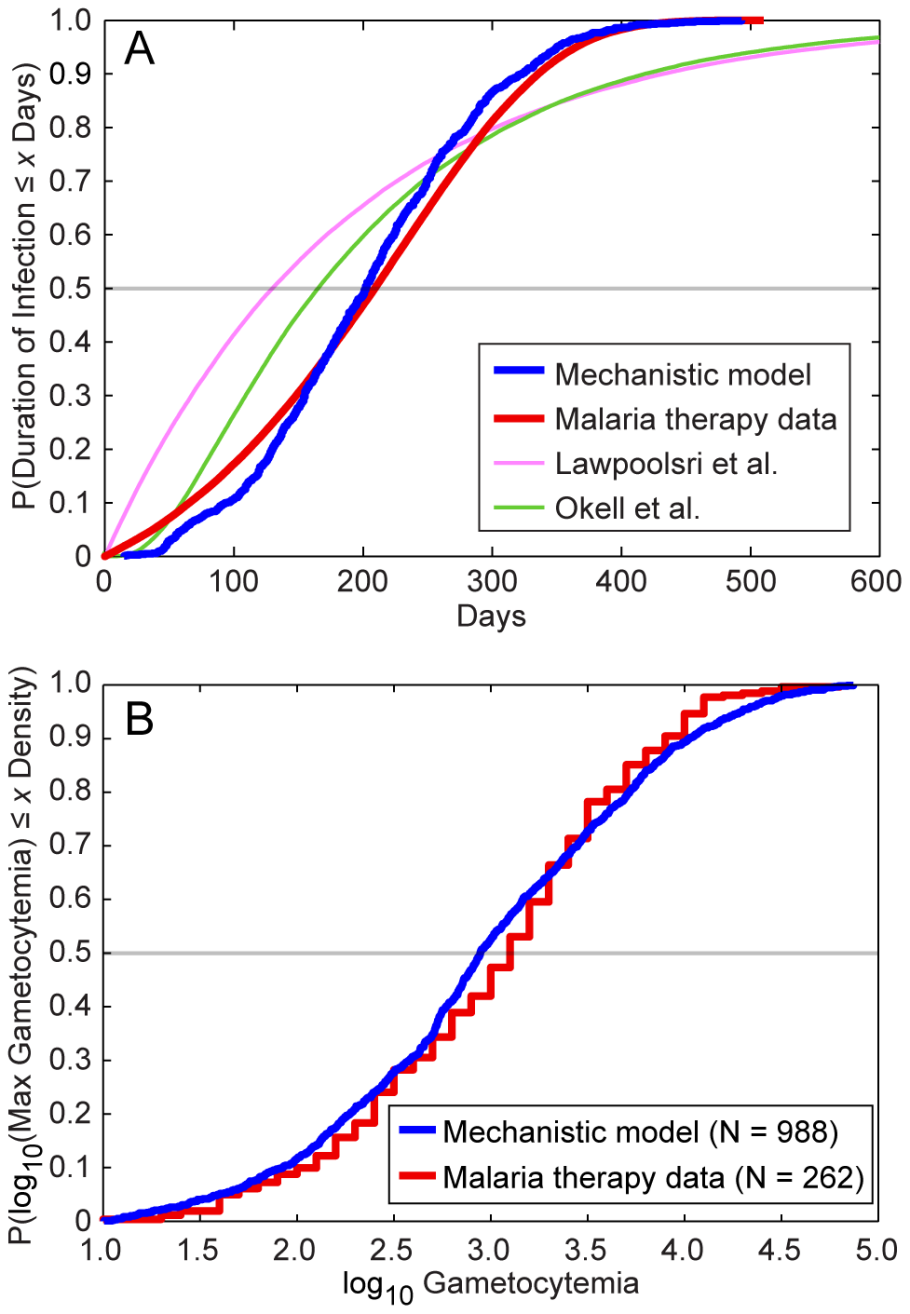
Comparison of model and malaria therapy cumulative distributions for two indices. (A) These line curves show the cumulative distributions of the durations of infection for the malaria therapy data, as well as those of our mechanistic model and the compartmental models of Lawpoolsri et al. [29] and Okell et al. [12]. The distribution from the malaria therapy data comes from fitting a Gompertz probability distribution to the durations of infection from 54 patients, as reported by Sama et al. [33]. The cumulative distribution function of the best-fit Gompertz distribution is plotted in red. The mechanistic model cumulative distribution was generated by calculating the durations of infection for 1,000 runs and plotting their empirical cumulative distribution function. The distributions from Lawpoolsri et al. and Okell et al. were generated by running those compartmental models according to their mathematical assumptions. The malaria therapy and mechanistic model distributions show relatively tight fits throughout the distribution. The durations of infections for the malaria therapy data and our mechanistic model are defined as the last observable day by smear minus the first observable day; the durations for the compartmental models are defined as the durations of time in infectious compartments. (B) We reviewed a total of 262 malaria therapy charts and recorded the maximum observed gametocytemia from each patient (data were recorded as log_10_ values to the nearest tenth) [24], [65]. We then recorded the maximum gametocytemias from 1,000 runs of our model. Because the malaria therapy data only include individuals who recorded at least four positive gametocyte observations, we censored out model runs in which gametocyte levels never exceeded 10 per µL (N = 988) [24]. Illustrated are the empirical cumulative distributions for the two data sets after log-transformation, i.e., the proportion of data that are less than or equal to a given level of log_10_ gametocytemia.

**Figure 4** provides a graphical illustration of two measures of model fit using the best-fit parameters. **Figure 4A** illustrates a measure of goodness-of-fit for our asexuals model, specifically the cumulative distributions of the durations of infection for both our model and the malaria therapy data [33]. The grey horizontal line illustrates the median durations of infection: our within-host model has a slightly shorter median duration (196 days) than the malaria therapy data (215 days) [33]. The slope of the cumulative distribution function from our model outputs is slightly steeper than that from the malaria therapy, indicating less variation in our modeled durations of infectivity compared to the malaria therapy data. However, the maximum durations of infectivity between model and malaria therapy are very similar. **Figure 4A** also shows the cumulative durations predicted by the two other models (in pink and green; see below).

For our model of gametocyte densities, we visually examined a total of 262 malaria therapy charts provided by Diebner et al. [24], [65] and recorded the maximum observed gametocytemia from each patient (data were recorded as log_10_ values to the nearest tenth). We then compared these data to the maximum gametocytemias from 1,000 runs of our model using the best-fit parameters. Because the Diebner et al. study only includes individuals who recorded at least four gametocyte-positive observations [24], we censored out model runs in which gametocyte levels never exceeded 10 per µL, leaving 988 runs remaining.

**Figure 4B** provides the empirical cumulative distributions of the durations of gametocytemia for the two data sets after log-transformation, i.e., the proportion of data that are less than or equal to a given level of log_10_ gametocytemia. The malaria therapy values are slightly higher on average initially, with a median of 3.10 for malaria therapy versus 2.95 from the model (grey horizontal line). Our model had a broader tail than the malaria therapy data, with more elevated gametocytemias than observed in the therapy data. The mean from the malaria therapy data was 3.01, whereas the mean from the model was 2.98. However, in our model, we estimated gametocytemias every day (i.e., we captured every maximum possible), as opposed to the sparser sampling of the malaria therapy data. Further, some of the individuals included in the patient charts from [24] were treated with chloroquine, chlorguanide, or quinine to terminate the infection after the initial period of continuous patent asexual parasitemia [23]. This treatment may have slightly biased downward the recorded malaria therapy maxima.

### Classical description of host contributions to *R_0_*

Once we were able to generate malarial infections *in silico* that resembled malaria therapy data across a variety of indices, we then attempted to quantify the distribution of human infectivity over time. The basic reproduction number *R_0_* is one of the most important parameters for quantifying the infectivity of a disease [15]. The classical expression for the *R_0_* of malaria was derived by Macdonald and can be formulated with four terms [46], [66], [67]. Potential transmission by a mosquito population is described by its vectorial capacity, *V_0_*, which describes the number of infectious bites that would arise from all the mosquitoes that bite one fully infectious individual on a single day. Two parameters, *b* and *c*, describe the proportion of blood meals that successfully cause an infection: *b* is the probability that an infected mosquito will infect an uninfected human upon biting; *c* is the probability than an infected human will infect an uninfected mosquito during a blood meal. In the Ross-Macdonald model, the infectious period of humans is exponentially distributed, with a daily clearance rate of *r* and a mean duration of infection of *r* ^−1^ days. The basic reproduction number of malaria is then described by the classic formula:
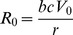
The Ross-Macdonald model [46], [66], [67] assumes that *c* is a constant over this period, so the ratio *c/r* describes the net infectiousness of a simple human infection. This net infectiousness fraction can be interpreted as the number of days that a person is fully infectious.

### Mean human infectivity over time

In reality, neither *V_0_*, *b*, *c*, nor *r* are constant among individuals over time and *R_0_* is only the first moment of a complicated multivariate distribution. Consider a population of *N* individuals, none of whom have been previously exposed to malaria. These individuals will differ in their responses to malarial infection, including onset of first fever relative to the initiation of blood stage infection, immune responses to asexual and sexual parasite densities over time, and the time to clearance of infection. We let *D_i_(t)* denote the probability that individual *i* will infect a mosquito upon being bitten at time *t*; this function takes values between 0 and 1. With our mechanistic model, one can simulate the full variability of *D_i_(t)* for populations with no acquired immunity.

**Figure 5:**
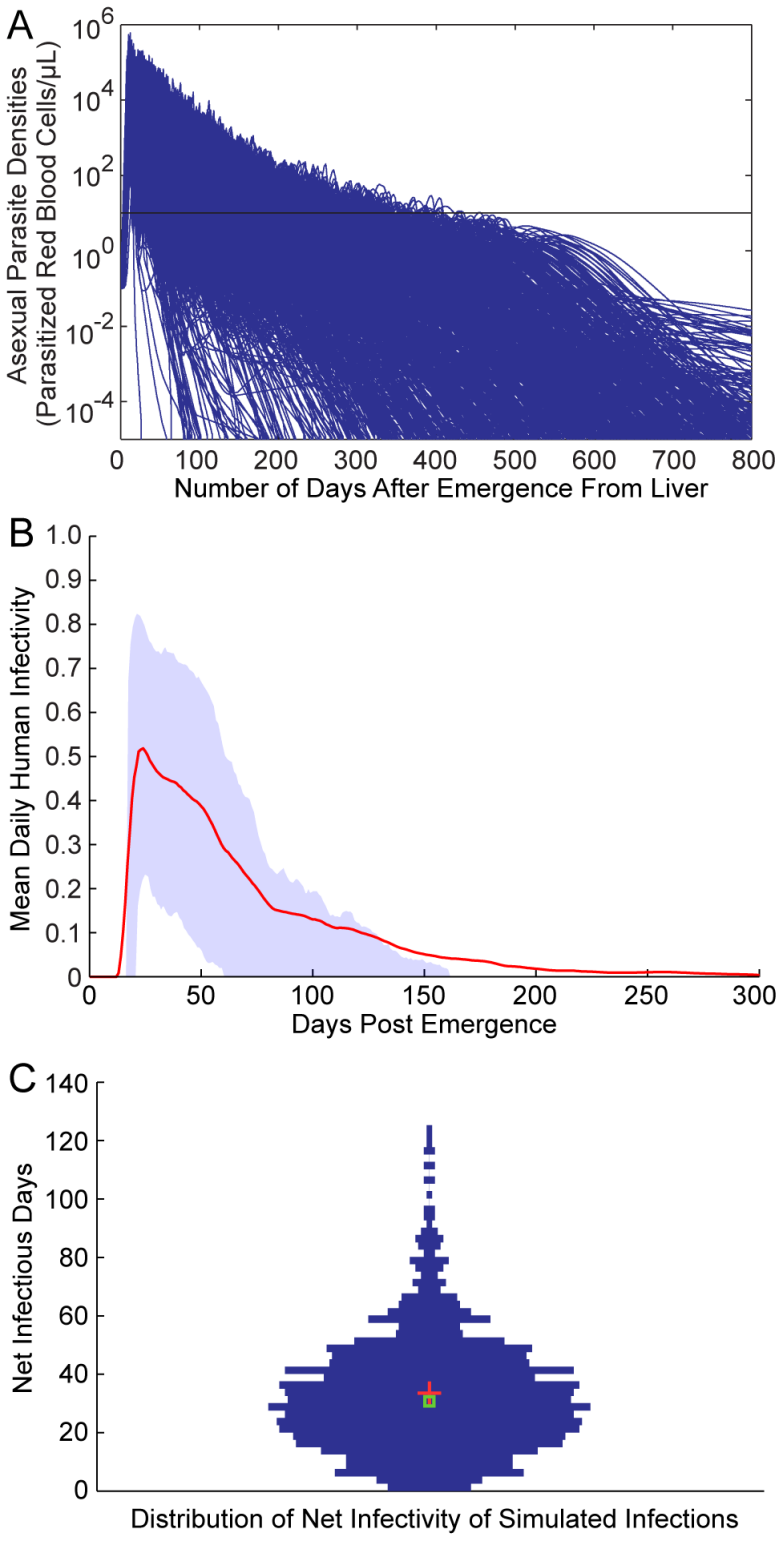
Mechanistic model predicted human infectivity over time and within a population. We calculated daily human infectivity to mosquitoes, as a function of time post emergence, for 1,000 simulated individuals. (A) Asexual parasitemias from 1,000 model runs. The wide diversity of host-parasite dynamics was fitted to malaria therapy data. (B) The mean daily infectivity of 1,000 simulated individuals for the first 300 days post emergence is shown as the red curve, and the area between the 25^th^ and 75^th^ daily infectivity percentiles is shown in blue. (C) Net infectivity for each of 1,000 individuals. The distribution of net human infectivity is represented as a violin plot, which extends to the maximum infectivity. The red cross illustrates the arithmetic mean infectiousness, and the green box shows median infectiousness.

If we first consider the mean of *D_i_(t)* within a population using the formula
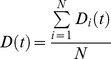
the resulting function *D(t)* is a function of time only. We call this function the mean human infectivity over time. Mean human infectivity is an important function for elimination in many contexts. Calculation of *D(t)* allows for a determination of how likely malaria will be able to persist through droughts or intensive antimalarial campaigns. The function *D(t)* for our mechanistic model is shown in **Figure 5** under best-fit model parameters. In **Figure 5A**, the simulated asexual parasitemias from 1,000 runs of the model are illustrated. A large diversity in responses can be observed, with asexual parasitemias differing among individuals by many orders of magnitude post emergence. These differences in asexual parasitemias are also mirrored in large differences among individuals in both gametocyte densities and human-to-mosquito infectivity over time (not shown). **Figure 5B** illustrates the 25^th^ and 75^th^ percentiles of daily infectivity for these simulated individuals, as well as the mean infectivity over time (in red). The mean infectivity *D(t)* is skewed due to the presence of some individuals exhibiting long-lived infectious periods. One important prediction from our model is that mean infectiousness is greater than five percent for 138 days after infection (see Discussion).

If we integrate *D_i_(t)* over time, rather than over individuals, we obtain
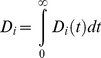
We call *D_i_* the distribution of net infectivity within a population. This distribution describes how individuals vary in infectiousness given the natural variability in host-parasite interactions. Our model-predicted *D_i_* is shown as a violin plot in **Figure 5C**. The infectivity of most individuals is clustered around the mean value (32 fully infectious days); however, there are an appreciable number of individuals who are predicted to be much more infectious than the mean individual. The maximum observed infectivity is 125.2 fully infectious days.

If we integrate either the mean human infectivity over time *D*(*t*) with respect to *t*, or the distribution of net infectivity *D_i_* over a population, we arrive at what we call the mean net human infectivity, *D*. The quantity *D* was first described in the supplement to [1]; this malaria map made use of preliminary results from the model described here. *D* can be calculated in one of two ways:
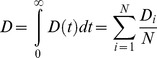
For our mechanistic model *D* ranges between approximately 31–34 when averaged over a population of 1,000 individuals (the mean of 5,000 runs was 32.4). The units of *D* can be considered as fully infectious days, i.e., the number of days in which an individual has a probability of 1 of infecting a mosquito. This value represents the human contribution to *R_0_*, and we note here that *D* is invariant across time, space and ecological setting.

### Comparison of infectivity over time and net infectivity among malaria models

Once we had computed *D_i_*, *D(t)*, and *D*, we then compared our calculations to values imputed from three other models: those of Lawpoolsri et al. [29], Okell et al. [12], or Dietz et al. (known as the ‘Garki model’) [28]. The former two models were designed to simulate the effectiveness of antimalarials at reducing malaria transmission and are the focus of our comparisons. The model of Lawpoolsri et al. was fitted to data from a low-transmission region of Thailand (P*f*PR∼0.0–1.5) [29] while the model of Okell et al. was fitted to three regions of medium intensity transmission in Tanzania. Both are compartmental models (Lawpoolsri et al. has one infectious compartment and Okell et al. has four infectious compartments varying in infectivity and clearance rate), and both papers employ their models to predict the constant equilibrium prevalence in untreated and treated cases.

We first compared the *D(t)* predicted from our model with those from Lawpoolsri et al. [29] and Okell et al. [12]. Lawpoolsri et al. assume that the mean rate of clearance in infectious individuals is 1/188 day^−1^ with a constant daily human-to-mosquito infectiousness (*c*) of 0.5. In the model of Okell et al. [12], each of the four infectious compartments in this model had different clearance rates (1/10.5, 1/10.5, 1/31.5, 1/157.5 day^−1^) and each compartment had a different proportional infectivity (1.90, 3.08, 1.53, 0.28) of the average daily infectivity *c* = 0.05. We did not weight these durations of infectivity for age or body surface area, i.e. we calculated the unweighted *D(t)*.

**Figure 4** illustrates the cumulative distributions of the durations of infection and infectiousness for these two models as well that of the mechanistic model. We see that our mechanistic model matches the malaria therapy curve closely compared to the compartmental models. These latter models have significantly heaver tails, indicating that individuals are infected for longer periods of time in those models.

**Figure 6:**
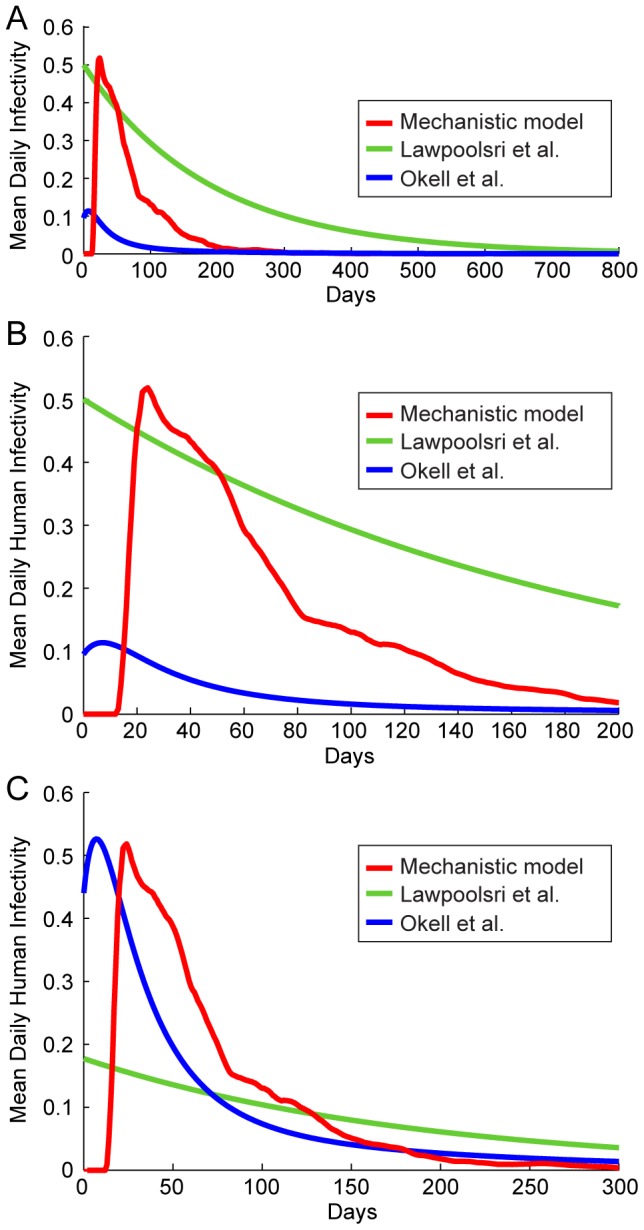
Comparison of mean infectivity over time, ***D(t)*** **.** The mean human infectivity to mosquitoes was calculated as a function of time for three models: our mechanistic model as well as the stochastic representations of the models of Lawpoolsri et al. [29] and Okell et al. [12]. For each model, the mean daily infectivity of 1,000 untreated individuals was simulated. (A) Mean infectivity for the first 800 days for the three models. (B) Mean infectivity for the first 200 days only. (C) Infectivity curves for the three models, scaled so that mean infectivity is equal to that of the mechanistic model.

We can derive *D(t)* for the compartmental models [12], [29], using the curves from **Figure 4A** and the *c* values for each compartment. **Figure 6A** shows *D(t)* for both of these models as well as our mechanistic model; **Figure 6B** illustrates the first 200 days of this function for closer inspection. We see that the model of Lawpoolsri et al. predicts that mean infectivity is above 5% for 433 days, the output from Okell et al. is above this threshold for only 45 days, and our mechanistic model output is above this value for 138 days (or until ∼153 days after emergence of parasites from the liver, discounting the initial period when infectivity is near zero).

The differences in *D(t)* among the models may have to do with model structure. Lawpoolsri et al. is constrained functionally by the assumption of only one infectious compartment. Okell et al. uses four infectious compartments and thus encompasses a much larger class of distributions (the hypoexponential distributions) for the lifetimes of infection. Further, by weighting the infectivity of each of the duration of infectiousness compartments differently, Okell et al. increase the degrees of freedom of *D(t)*, allowing them to more closely fit their target data. Further, these two models differ in the data sets being fitted: the endemicity of the regions being modeled at equilibrium in Lawpoolsri et al. are much lower than those in Okell et al. It is possible that individuals in low-endemicity areas are infectious at higher levels for longer periods than individuals in high-endemicity areas, because acquired immunity may limit the severity and density of repeated *P. falciparum* infections. This effect may provide a means of identifying the effects of immunity on transmission. However, we would need to fit a variety of endemic equilibria with hypoexponential models such as that of Okell et al. to test such a hypothesis. We cannot generate quantitative conclusions from comparing the models of Lawpoolsri et al and Okell et al directly, given their different model structures.

Integrating over time, we find that the *D* values for these three models are 7.2 fully infectious days for the model of Okell et al., ∼32 days using the current model, and 94 days in the model of Lawpoolsri et al. We can also compare these values to an older field-tested compartmental model, known as the ‘Garki model’ because it was fitted to data from a malaria-endemic site in Garki, Nigeria [28]. This model includes compartments for immunity such that immune individuals clear infections faster than non-immune individuals.

To calculate the net human infectiousness *D* for this model, let *V_0_* be the vectorial capacity of an area. For malaria, 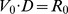. Further, let ***V*** be the critical vectorial capacity below which transmission is unstable, i.e., 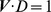. Thus, *D* = 1/***V***. As derived in the Garki model, 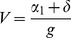, where *α*_1_ is the clearance rate of infectivity, *δ* is the death rate, and *g* is the probability of becoming infected by the bite of an infected mosquito; thus 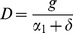 [28]. Using the values derived from Garki, we find that *D* = 45.5 fully infectious days. For the Garki estimate, the values of *α*_1_ and *δ* were assumed and only *g* was fitted to data; thus essentially *D* itself was fitted to data as a single parameter [28]. This fitted value for *D* accords relatively well with the value generated by our model [28].

Given our calculations of *D*, we can rescale the plots of *D(t)* by multiplying each curve by a scaling factor so that models of Lawpoolsri et al. and Okell et al. share the same mean net infectivity as the mechanistic model; these results are shown in **Figure 6C**. Once the models are rescaled, we can see more clearly that the models of Okell et al. and the mechanistic model predict that infectiousness is cleared at very similar rates throughout the population, whereas Lawpoolsri et al. predict a much more gradual loss of infectiousness. The closeness of *D(t)* for the scaled stochastic representation of Okell et al. and the mechanistic model is somewhat surprising, although Okell et al. do parameterize some of their model parameters from malaria therapy data.

### Comparison of individual variability in human-to-mosquito infectiousness among malaria models

**Figure 7:**
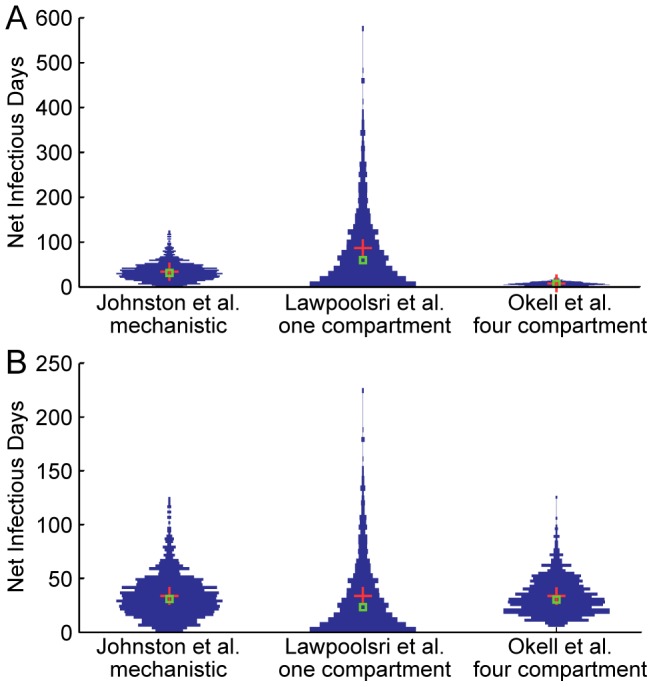
Comparison of distributions of net human infectivity, ***D_i_*** **.** The distributions of net human infectivity were calculated for three models: our mechanistic model as well as the compartmental models of Lawpoolsri et al. [29] and Okell et al. [12]. (A) The infectivity for each of 1,000 individuals was integrated over time for each model. The distributions of net infectivity among individuals are represented as violin plots (vertical histograms); the plots extend to the maximum infectivity. (B) Scaled distributions of net infectivity. The distributions in panel (A) were rescaled by multiplying by a scaling factor such that all three distributions had the same mean as that of the mechanistic model. The red crosses illustrate the arithmetic mean infectivity, while the green boxes show the median infectivity.

In the previous section we calculated the mean responses of individuals over time for the models of Lawpoolsri et al. [29] and Okell et al. [12]. However, since these models are both compartmental, they can readily be formulated as stochastic, individual-based models by assuming that individuals are in each infectious compartment for exponentially distributed times. We thus computed the distribution of net infectiousness within a population, *D_i_*, for both models. **Figure 7** compares the distributions *D_i_* for these two compartmental models to the distribution generated by our mechanistic model. As implied by the *D(t)* curves, **Figure 7A** illustrates that the model of Lawpoolsri et al. predicts that some individuals have very high *D* values, whereas the distribution *D_i_* generated by the model of Okell et al. is much more centered about its mean. If we scale the distributions *D_i_* to all have the same mean as the mechanistic model, we see that *D_i_* for Lawpoolsri et al. is still much more dispersed than the mechanistic model; however, *D_i_* for Okell et al. matches quite well to that of the mechanistic model (**Figure 7B**).

### Sensitivity analyses

We ran a variety of sensitivity analyses by varying the model parameters and observing the changes in model output. For the asexuals model, we adjusted 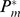 such that the mean duration of infection varied between 183 and 237 days (∼95% confidence interval as reported by Sama et al. [59]). We found that the net infectivity for the model varied from 29.9 to 37.4 net infectious days, versus 32.4 for the best-fit parameters [1].

For the gametocyte model, we examined the effects of varying the *α_G_* parameter. For our best-fit parameterization, we assumed that *α_G_*∼*U*(0.06, 1). If we assumed that *α_G_* followed the *U*(0, 1) distribution, then the maximum average circulation time increased to 24.0 days (close to the 22 recorded in malaria therapy; **Table 3**). The average maximum circulation time was increased because the lower bound of the uniform distribution was changed from 0.06 to 0.0, i.e., in some individuals gametocyte age had no effect on gametocyte longevity. The average infectivity of the population was increased by a small amount to 34.6 using the wider bounds for *α_G_*, versus 32.4 for the model with *α_G_*∼*U*(0.06, 1). Further, the maximum number of net infectious days for *α_G_*∼*U*(0, 1) was 181.5, versus 125.2 for *α_G_*∼*U*(0.06, 1). Thus, *α_G_*∼*U*(0, 1) produced a very heavy tail in the distribution of infectivity among individuals.

Regarding the relationship between gametocyte density and human-to-mosquito infectivity, our default model outputs assumed the relationship from Stepniewska et al. as fitted from malaria therapy [48]. We also simulated the effects of assuming different types of gametocyte density to infectivity relationships. Specifically, we simulated 14 different types of possible functional relationships between gametocyte densities and infectivity (**Figure 3**). Our default assumption was called the ‘Median, Stage V’ relationship (solid red line in **Figure 3**); we also assumed both ‘High’ and ‘Low’ Stage V relationships (illustrated as dashed red lines in **Figure 3**). These latter relationships were chosen to capture much of the observed variation in the Carter and Graves data [63], [64]. Further, we ran a logistic regression through the Carter and Graves data to develop another functional relationship (dark blue line in Figure 3); this logit fit was similar to the data reported in the meta-analysis of Bousema et al. [56]. Each of these four relationships relates observable (Stage V) gametocytes to infectivity, and for each of these four relationships we could apply the Jeffery-Eyles observation that gametocytes are not infectious at the onset of gametocyte appearance [47] to generate a total of eight density-to-infectivity relationships.

To develop the six other possible relationships between gametocyte densities and infectivity, we utilized additional information regarding the biology of *P. falciparum*. Not all gametocytes that are observable are infectious; once gametocytes enter the circulation, they still need a brief number of days to mature further before becoming infectious [47], [68], [69]. Circulating Stage V gametocytes can be further discriminated into two categories: Stage VA gametocytes and Stage VB gametocytes [68]. Stage VA gametocytes are circulating but are not infectious; Stage VB gametocytes are both circulating and infectious. Thus we generated three additional functional relationships by assuming that observable gametocytes were infectious only after two additional days of maturation. These three relationships were designed to parallel the ‘Median,’ ‘High,’ and ‘Low’ relationships from above but assuming only Stage VB gametocytes are infectious; these are illustrated in light blue in **Figure 3**. We then modified each of the three Stage VB assumptions by assuming that there is a short period at the beginning of infections in which gametocytes are not infectious, as above [47], for a total of 14 possible functional relationships between gametocyte density and infectivity.

The mean net infectivity values for seven of the parameterizations are 70.0, 41.1, 23.6, 64.1, 33.3, 16.2, and 36.3 net infectious days for the Stage VB, High; Stage VB, Median; Stage VB, Low; Stage V, High; Stage V, Median; Stage V, Low; and Carter & Graves parameterizations, respectively (without the Jeffery-Eyles corrections and with *mintrans* = 0). If we include the effects of the Jeffery-Eyles correction, these seven parameterizations yield 68.2, 40.2, 23.1, 61.2, 32.0, 15.6, and 35.0 mean net infectious days, respectively (assuming *mintrans* = 0). Each mean is from 1,000 runs. Varying the assumed relationship between gametocyte density and infectivity will also affect other aspects of transmission by altering the duration between parasite emergence and infectivity and/or the total duration of positive infectivity.

Also of note, our model calculated *P. falciparum* infection dynamics only among adults, as there are no malaria therapy data for children and it is not well-understood how children differ in their overall levels of infectivity from adults. In a companion paper (Johnston et al., *in prep*) we discuss how our results concerning infectivity among adults may translate to children and the implications of using our model results for malaria control planning.

## Discussion

Here we describe the development of a novel, stochastic, within-host model of the progression of malaria in patients with no acquired malarial immunity. This model utilizes the difference equations originally developed by Molineaux and Dietz to simulate the progression of asexual and sexual parasitemias [23]–[25]. We have parameterized these equations so that the entire range of observed responses in malaria therapy can be reproduced without needing to fit parameters to individual case histories. We also extended the modeling framework from [23]–[25] to include components for simulating the onset of first fever and human-to-mosquito transmission.

Once our mechanistic model was formulated, we revisited the analytic Ross-Macdonald model to examine how human infectiousness enters into the formula for the basic reproduction number *R_0_*. We then analyzed human infectiousness in three ways, calculating the mean human infectivity over time *D(t)*, the distribution of net infectivity *D_i_*, and the mean net human infectivity, *D*. We found that *D* in our mechanistic model is approximately 32 fully infectious days. This quantity is invariant in a population over time and plays a crucial role in determining *R_0_*. We have utilized this value in recent malaria mapping work [1], although a full mathematical treatment of this quantity was left until the present.

Our study included a review of the mathematical literature to determine whether we could impute these quantities from other modeling work to provide a baseline for comparison. We examined the models of Lawpoolsri et al. [29], Okell et al. [12], and the Garki model [28], and found them to vary widely in their calculation of *D*, *D(t)* and *D_i_*. We propose that our new estimate of *D* is the most appropriate one for *R_0_*, because *R_0_* assumes no acquired immunity and our model is parameterized solely from malaria therapy studies with individuals that were non-immune. The other models cannot easily disentangle the effects of acquired immunity, multiplicity of infection, and control efforts from the effects of immunity acting on a single infection, though we have described how future efforts might begin to separate these quantities.

In addition to our calculation of the invariant *D* and its importance for *R_0_*, we also predict that human infectiousness persists for a long period of time at levels sufficient to promote transmission in areas of high vectorial capacity. While these calculations are for populations with no acquired immunity, they are relevant for malaria elimination efforts because antimalarial immunity wanes rapidly in the absence of infection [17], [70]. As this immunity wanes, the responses of individuals to infection can be expected to approach those observed in malaria-naïve individuals [28], [66]. Of note, a recent study in Senegal found that persistent infectiousness prevented interruption of transmission even when incidence had been reduced to very low levels through insecticide-treated bed nets and usage of ACTs [17]. Our model confirms the relevance of persistent low-level infectiousness for elimination efforts.

In addition to the usefulness of these results for mapping and control efforts, the modeling platform and analytic framework described herein will help clarify the different assumptions among malaria models. Further, because we calculate asexual and sexual parasite densities daily, and because the model reproduces the entire variability of host-parasite dynamics observed in malaria therapy, our modeling framework provides a powerful new tool for exploring the effects of antimalarial treatments on transmission. As malaria decreases worldwide, our model results will become more relevant to more regions of the world, thus helping to improve targeting of control efforts.

## Supporting Information

Dataset S1 Standalone model designed for Windows operating systems.
(ZIP)

Dataset S2 Standalone model designed for Macintosh operating systems.
(ZIP)

Dataset S3 Source code for malaria model and graphical user interface components.
(ZIP)

Figure S1 Graphical user interface of standalone model software.
(PDF)

Text S1 User guide for use of the model software.
(PDF)
