# Supplementary material for: Malaria's Missing Number: Calculating the Human Component of R0 by a Within-Host Mechanistic Model of Plasmodium falciparum Infection and Transmission
Source: PLoS Comput Biol. 2013 Apr 18;9(4):e1003025. doi: 10.1371/journal.pcbi.1003025 (PMC3630126; doi:10.1371/journal.pcbi.1003025)
Supplement: Figure S1 — Graphical user interface of standalone model software. (PDF) [file pcbi.1003025.s004.pdf]

Figure S1

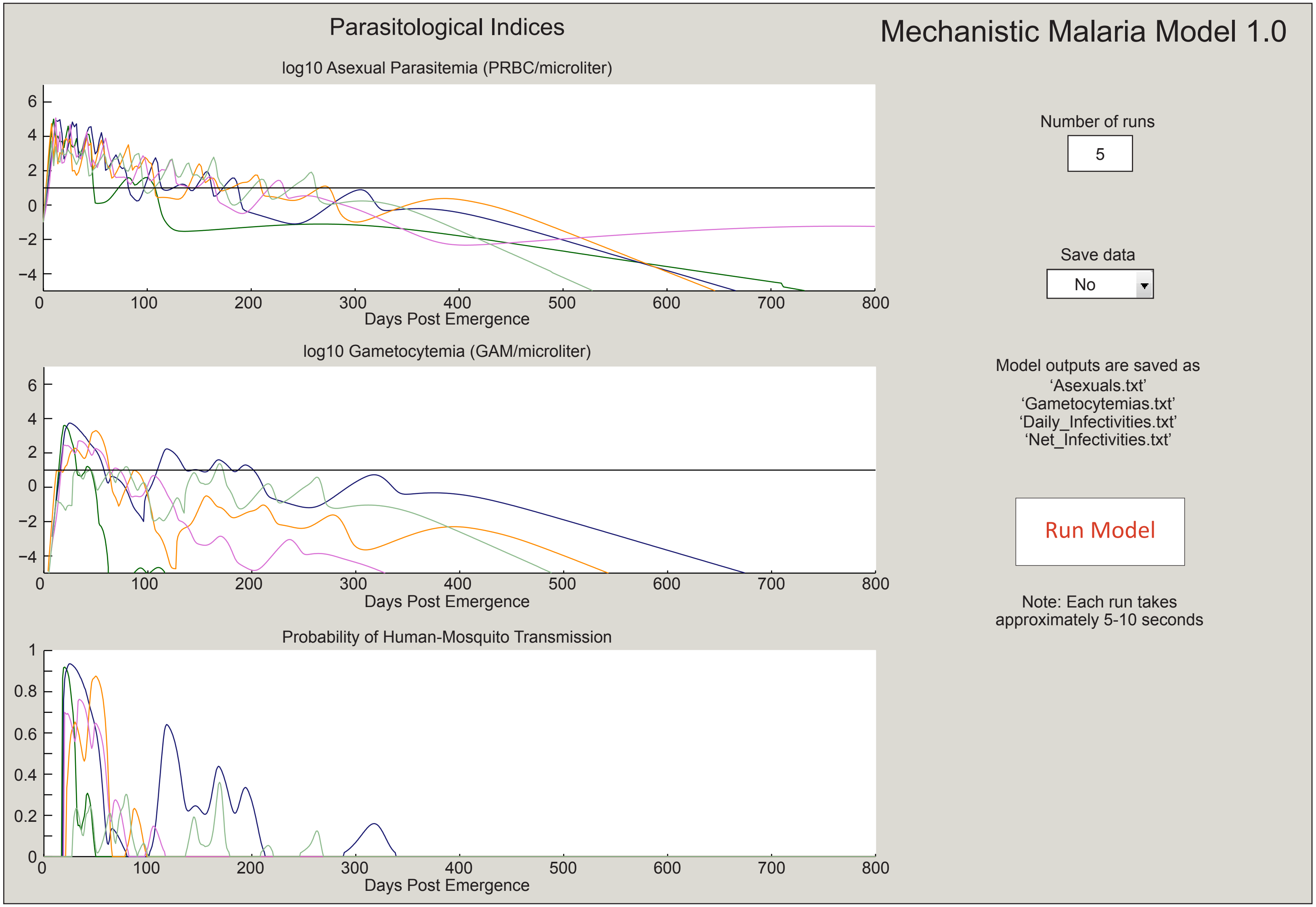

Graphical user interface for standalone model software. User manuals for PC and Mac are provided as Supplemental Text S1. Model files are also included.
