# Supplementary material for: Malaria's Missing Number: Calculating the Human Component of R0 by a Within-Host Mechanistic Model of Plasmodium falciparum Infection and Transmission
Source: PLoS Comput Biol. 2013 Apr 18;9(4):e1003025. doi: 10.1371/journal.pcbi.1003025 (PMC3630126; doi:10.1371/journal.pcbi.1003025)
Supplement: Text S1 — User guide for use of the model software. (PDF) [file pcbi.1003025.s005.pdf]

Supplemental Text S1: User manual for standalone version of model

A Missing Number for Malaria: Calculating the Human Component of R_0 with a Mechanistic Model of *Plasmodium falciparum* Infection and Transmission

Geoffrey L. Johnston, David L. Smith, and David A. Fidock

I) Installation
   a. On a Windows computer
   b. On a Macintosh computer

II) Running the Model

III) Saving the Outputs

IV) For Researchers

V) Terms of use
   a. Redistribution, modification, and/or utilization for commercial purposes

I) Installation

a. On a Windows computer

If you do *not* have MATLAB, you need to install a program called MCRInstaller that allows execution of MATLAB code (.m files) without owning a copy of MATLAB. The software is located at: http://www.mathworks.com/products/compiler/mcr/. Be sure to download and install the program for **Release R2012b** (the 'Typical' installation is recommended).

The second file that you will need is the model file itself (***PCmodel.zip***). Place this file in the same directory as the MCRInstaller and extract the package contents. To run the M^3 software, simply double click on ***malaria_model.exe***; this should open the graphical user interface to the model.

If you *do* have MATLAB, you can use the source .m files (***model_GUI_1_0.m*** and ***final_model.m***) to run the software. The code was developed in version R2012b; the software may not run correctly in other versions of MATLAB. Be sure to unpack all of the source code into the same folder.

b. On a Macintosh computer

If you do *not* have MATLAB, you need to install a program called the MCRInstaller that allows execution of MATLAB without owning a copy of MATLAB. The software is located at: http://www.mathworks.com/products/compiler/mcr/. Be sure to download and install the program for **Release R2012b** (the 'Typical' installation is recommended).

The second file that you will need is the model file itself (***MACmodel.zip***). Extract this file to a directory of your choice. To run the M^3 software, follow the steps listed in the ***readme.txt*** file. To run the file, you will likely need to open a Terminal window, navigate to the directory in which the file ***run_malaria_model.sh*** is located, and then type 'sh run_malaria_model.sh <dir>', where <dir> is the directory where you installed the MCRInstaller.

If you *do* have MATLAB, you can use the source .m files (***model_GUI_1_0.m*** and ***final_model.m***) to run the software. The code was developed in version R2012b; the software may not run correctly in other versions of MATLAB. Be sure to unpack all of the source code into the same folder.

II) Running the Model

To run the model, first choose the number of simulations to run, and whether you want the data to be saved. The 'Save data' parameter must be set before the simulation begins (see section below). Once the settings are chosen, click on the 'Run Model' box. The model will then begin simulations. Two progress bars will open, the first titled 'Asexual Calculations' and the other 'Gametocyte Calculations.' These two progress bars track the simulations as they are being run. Both progress bars must indicate 100% progress before the simulations are complete.

Please note that the source code for the model is located in the file ***final_model.m***. The files ***model_GUI_1_0.m*** and ***model_GUI_1_0.fig*** are files used to generate the model's graphical user interface (GUI). The file ***progressbar.m*** is used to generate the progress bars in the GUI and was written by Steve Hoelzer.

Also, in the model source code (***final_model.m***) there are sections that refer to unsupported model functionality. We are currently adding additional features to the model that are not supported in Version 1.0 but which we hope will be supported in future versions.

III) Saving the Outputs

The 'Save data' parameter needs to be set to 'Yes' *before* the model is run; you cannot save data by adjusting this parameter once the model has started. Assuming that 'Save data' is correctly set and the model run, the model will save the following outputs: the daily asexual parasitemias, the daily gametocytemias, the daily infectivities, and net infectivities for each run. Each of these four sets of data is saved to a separate ASCII text file. The file names are '**Asexuals.txt**,' '**Gametocytemias.txt**,' '**Daily_Infectivities.txt**,' and '**Net_Infectivities.txt**.' These files should appear in the directory in which the model is run.

IV) For Researchers

Although the model GUI provides functionality to the end-user, researchers may desire additional capabilities. For example, researchers may desire to modify parameters beyond their default values in the GUI or run very large numbers of simulations.

To perform the simulations in the paper, as well as ongoing work, the authors use a high-performance computing cluster known as Hotfoot, based at Columbia University. To access this cluster, the authors wrote a script for Maui/Torque that allows for parallel execution of the model and modified the MATLAB code so that the code can be called by this script. This cluster allows for hundreds of simultaneous runs, such that the time needed to run simulations is reduced many-fold versus single-instance simulation. If researchers are interested in accessing this resource to run their own simulations, please contact the corresponding authors for more information.

V) Terms of use

You agree to all of the following terms of use when redistributing/modifying/using this software in any way, including use of its output and/or any part of its source code:

Redistribution, modification, and/or utilization for commercial purposes

The Mechanistic Malaria Model (M^3) Simulator. Copyright © 2013 Geoffrey Johnston.

This program is free software: you can redistribute it and/or modify it under the terms of the GNU General Public License as published by the Free Software Foundation, version 3.

This program is distributed in the hope that it will be useful, but without any warranty; without even the implied warranty of merchantability or fitness for a particular purpose. See the GNU General Public License for more details.

For a copy of the GNU General Public License see http://www.gnu.org/licenses/.
